# Autism diagnosis in females by eating disorder professionals

**DOI:** 10.1186/s40337-023-00785-0

**Published:** 2023-05-11

**Authors:** Marissa A. Parsons

**Affiliations:** Recovery and Wellness Center of Eastern Washington, 1950 Keene Rd Bldg G, Richland, WA 99336 USA

**Keywords:** Feeding and eating disorder, Autism, Female, Adolescent, Diagnosis

## Abstract

**Importance:**

Autism and eating disorders cooccur at high rates, with autism impacting the efficacy of eating disorder treatments and outcomes. Females are underdiagnosed with autism and diagnosed later in life than their male counterparts.

**Objective:**

The purpose of this study was to define the incidence of eating disorder professionals identifying autism in female adolescents and young adults engaged in treatment for an eating disorder.

**Design:**

The research design is a cross-sectional retrospective records review. The charts reviewed were from the medical records of forty assigned-at-birth females between the ages of 13–25 who received treatment for an eating disorder at the partial hospitalization program (PHP) level of care between 2020 and 2022.

**Main outcomes:**

Upon entering PHP for an eating disorder, 10% of the study participants had a pre-existing diagnosis of autism. A total of 27.5% of participants had clinical suspicion of autism. The number of autism traits that an individual possessed directly correlated with the number of calendar days in treatment.

**Conclusion:**

Eating disorder professionals in this study identified autism in 17.5% of adolescent and young adult females who entered PHP treatment for an eating disorder who had not previously been diagnosed with autism. Eating disorder professionals can anticipate that individuals with autism may be in treatment for a longer duration. Further studies should explore effective treatment measures for those with autism in treatment for an eating disorder.

## Abstract (summary)

It is well established that autism and eating disorders often co-occur. Females are less likely to be diagnosed with autism and tend to receive a diagnosis later in life than males. Therefore, during eating disorder treatment at higher levels of care, eating disorder professionals may be the first to recognize autism in their female patients. A review of chart records from female teens and young adults receiving eating disorder treatment at the partial hospitalization level of care between 2020–2022, revealed that 10% had a diagnosis of autism upon entering treatment and an additional 17.5% received a new diagnosis during treatment. The number of autism traits present in an individual directly correlated to longer treatment durations.

## Introduction

The link between autism and eating disorder was first described in 1983 by Swedish child and adolescent psychiatry professor Christopher Gillberg. He observed that three boys with autism had female cousins with an eating disorder and hypothesized that anorexia nervosa (AN) was the female version of autism [1]. Once thought to be a male-dominant diagnosis, autism has shifted to a more realistic gender ratio of 3:1 [2] in recent decades and affects one in 44 children [3].

However, autism is over-represented in individuals receiving treatment for an eating disorder, with co-occurrence rates of 23–32% [4, 5]. Potential autism-specific mechanisms underlying eating difficulties include deficits in social interaction and relationships, poor sense of self and identity, difficulties with emotions, and a need for control and predictability [6]. Additionally, individuals with autism have higher rates of picky eating, feeding problems, gastrointestinal symptoms, and sensory issues [7].

Females with autism continue to be underdiagnosed and diagnosed later in life than their male counterparts [8, 9]. Theories about the underdiagnosis in females include that the *Diagnostic and Statistical Manual, Fifth Edition* (DSM-5) may favor male-typical presentations, females are more likely to “mask” or “camouflage” their autism traits, or there is a gender bias in diagnosis even when the diagnostic criteria are met [10]. A functional MRI study of males and females with autism revealed that their brains are functionally organized differently, contributing to their clinical symptoms in distinct ways [11].

In the United States, one in five females develops an eating disorder by the age of 40 [12].

A 2018 longitudinal study evaluated the hypothesis that starvation, due to prolonged restriction, can induce autism traits that would resolve with adequate nutritional rehabilitation. However, the authors demonstrated that autism traits and diagnosis persisted after 12 months of treatment [13]. There is, therefore, a benefit to identifying autism in individuals with an eating disorder early in the treatment course. Individuals with an eating disorder and autism are more likely to have difficulties with cognitive behavioral therapy and group therapy, which are eating disorder treatment standards of care [14]. Additionally, these individuals have poorer outcomes regarding their eating disorder recovery and prognosis of mental health outcomes and socioeconomic function [13, 15]. These findings underscore the importance of identifying autism in individuals with an eating disorder as a prognostic factor.

Partial hospitalization treatment for an individual with an eating disorder is often facilitated as a day program through a multidisciplinary team of professionals that interact with their patients intensively for more than forty hours per week, across the span of several months. Therefore, eating disorder professionals may be the first clinicians to identify autism traits in their patients, particularly females. The purpose of this study was to define the incidence of eating disorder professionals identifying autism in female adolescents and young adults engaged in treatment for an eating disorder.

The article most closely related to this research study was a 2020 review article in which Brown and Stokes [16] discuss the reasons why eating disorder professionals may be the first providers to recognize autism in their female patients. Additionally, the authors reviewed autism and eating disorder comorbidity, treatment, and priorities for research and clinical practice. However, no original research exists on the subject. In a 2021 mixed-methods study of females with autism, parents of teenagers with autism, and their health care providers, Babb et al. [17] had an “unexpected finding” that all adult and adolescent females had received their autism diagnosis after they engaged in eating disorder services. The average age of AN diagnosis in the group was 17, but the average autism diagnosis was 29 years old. Similarly, in a semi-structured interview study of individuals with AN and autism, the majority of participants diagnosed with autism received their diagnosis after receiving eating disorder treatment, with the average age of diagnosis being 23.5 years of age [18].

In a large retrospective study, individuals with AN between the ages of 8–32 and matched controls, non-eating disorder diagnoses were monitored for a median of nine years [19]. The non-eating disorder diagnoses evaluated were substance use disorders, schizophrenia or psychosis, affective disorders, phobia or anxiety disorders, obsessive–compulsive disorders, adjustment disorders, personality disorders, and autism. For the individuals diagnosed with AN, 25% received at least one of those non-eating disorder diagnoses after two years [19]. At 20 years, 55% had received a non-eating disorder diagnosis [19]. At the onset of the study, 0.6% of the individuals with AN had a pre-existing diagnosis of autism (vs. 0.2% of the controls). The follow-up data at all time intervals yielded too few autism observations to be statistically significant. Therefore, the authors did not include follow-up data in the findings.

Currently, a gap exists in the literature defining the incidence of a new autism diagnosis in adolescent or young adult females after engaging in treatment of an eating disorder. To this author’s knowledge, no original research has been conducted on this topic, nor has the literature grossly estimated this incidence. In the present study, this author aimed to answer the following research question: “What is the incidence of new or suspected autism diagnosis in female adolescents and young adults after initiating eating disorder treatment?” Additionally, this study identified the risk factors associated with a new autism diagnosis and evaluated the impact of the presence of autism traits on treatment outcomes. It was hypothesized that the presence of autism in the general population.

## Methods

### Participants

The research design was a cross-sectional retrospective records review. The charts reviewed were from the medical records of 40 individuals that concluded treatment for an eating disorder at the Recovery and Wellness Center of Eastern Washington at the partial PHP level of care between 2020 to 2022. Eligible study participants were assigned-at-birth-females between the age of 13 and 25 at the time of PHP admission for an eating disorder that met the *DSM-5-TR* [20] criterion of AN, bulimia nervosa, binge eating disorder, atypical anorexia nervosa, avoidant-restrictive food intake disorder, purging disorder, or unspecified feeding and eating disorder.

The Recovery and Wellness Center of Eastern Washington’s PHP level of care is a day program run 8.5 h per day, five days per week. Determination for this level of care is made in accordance with the American Psychological Association’s eating disorder level of care guidelines. A transition from PHP to the intensive outpatient program (IOP) level of care is made at the recommendation of the individual’s treatment team once the individual has met treatment goals related to reduction of eating disorder behaviors. The IOP level of care is four hours a day three to five days per week.

### Data collection

Patient age, ethnicity, and eating disorder diagnosis at the time of admission were collected from the patient charts. The presence of co-morbid mental health conditions from the medical discharge summary were collected, including major depressive disorder, generalized anxiety disorder, post-traumatic stress disorder, obsessive compulsive disorder, attention deficit hyperactivity disorder, and mood disorder. Mood disorder for the purposes of this study includes bipolar I disorder, bipolar II disorder, disruptive mood dysregulation disorder, and cyclothymic disorder.

The presence of a historical diagnosis of autism, family history of autism, family history of autism in a first-degree relative, and history of developmental delays was collected from the intake medical assessment interview of the patient and a parent or guardian of minors. Autism traits for the purposes of this study are the individual components of the diagnostic criteria for autism per the *DSM-5-TR* [21], including deficits in social-emotional reciprocity, deficits in nonverbal communicative behaviors, deficits in developing, maintaining, and understanding relationships, stimming, rigidity, hyper-fixated interests, and sensory processing disorder. The presence of these traits was collected from chart notes from the medical provider and psychotherapists throughout the course of treatment.

The clinical suspicion of autism, referral for neuropsychological testing, and a new autism diagnosis was included from medical chart notes. The number of days in treatment at the PHP and intensive outpatient program level of care and the total number of calendar days from admission to discharge is reported. Individuals that were engaged in treatment for less than 15 calendar days were excluded, due to less clinician opportunity to observe autism traits. Whether or not the patient has previously been engaged in a higher level of care (residential, PHP, or IOP) for an eating disorder or whether they were a step-down from a residential treatment center to PHP was collected from the admission assessment.

The type of discharge is noted as graduation, discharge, or completion. A designation of graduation indicates that a patient met all expected treatment goals. Completion indicates that a patient reduced eating disorder behaviors and was appropriate for a transition to an outpatient level of care but did not meet all standards of treatment. A discharge refers to either a patient self-discharging from either PHP or IOP or discharged by the facility’s staff at any point in treatment.

### Statistical analysis

With the assistance of a statistician, the data sets were run through IBM SPSS software. A one-sample proportions test with Jeffreys 95% CI was run on the sample of 40 patients to answer the primary research question to determine if female adolescents and young adults receiving treatment for an eating disorder will have a higher prevalence of pre-existing, clinical suspicion, and new autism diagnosis than the general population.

An independent-samples *t*-test was run to determine if there were different percentages of autistic traits exhibited between the two groups. There were no outliers in the data, as assessed by inspection of a boxplot. The homogeneity of variances was assessed by Levene's test for equality of variances. A bivariate analysis using chi-square tests for association was conducted between family history of autism and autism variables. The homogeneity of variances was assessed by Levene's test of homogeneity of variances and a one-way ANOVA was conducted to determine if the percentage of autism traits exhibited were different between the three graduation groups.

## Results

### Descriptive characteristics

The study participants’ information was assessed using descriptive statistical techniques. Frequencies (*n*) and percentages (%) of the sample characteristics are presented in Table 1.

**Table 1:** Descriptive Characteristics

Variable	n	%
White	29	72.5
Hispanic	10	25
Asian	1	2.5
13–15 years	17	42.5
16–18 years	12	30
19–21 years	4	10
22–25 years	7	17.5
Anorexia nervosa	11	27.5
Atypical anorexia nervosa	16	40
Bulimia nervosa	5	12.5
Avoidant restrictive food intake disorder	3	7.5
Binge eating disorder	1	2.5
Purging disorder	2	5
Unspecified feeding or eating disorder	2	5
Previous eating disorder treatment in higher level of care	4	10
Step-down from a residential treatment center	7	17.5
Graduation	18	45
Discharge	11	27.5
Program completion	11	27.5
Depression	16	40
Anxiety	23	57.5
Post traumatic stress disorder	5	12.5
Obsessive compulsive disorder	3	7.5
Attention deficit hyperactivity disorder	9	22.5
Other mood disorder	10	25
Historical diagnosis of autism	4	10
Family history of autism	11	27.5
First degree relative with autism	5	12.5
History of developmental delays	8	20
Deficits in social-emotional reciprocity	13	32.5
Deficits in nonverbal communicative behaviors	20	50
Deficits in developing, maintaining, an understanding relationships	21	52.5
Stimming	4	10
Rigidity	16	40
Special interests	7	17.5
Sensory processing disorder	11	27.5
Clinical suspicion of autism	11	27.5
Referral for neuropsychological testing	4	10
New autism diagnosis	5	12.5

### Autism in eating disorder treatment versus general population

**Fig. 1:**
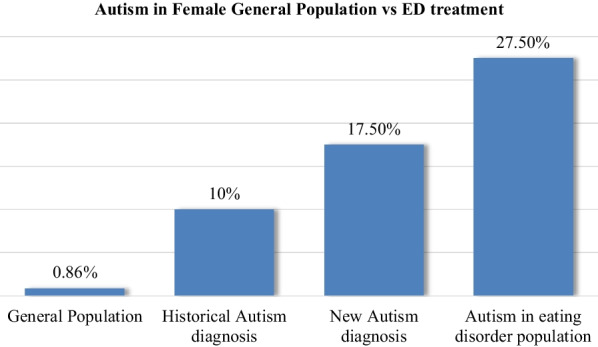
Autism in female general population versus eating disorder treatment

A historical diagnosis of autism upon admission to PHP was 10% (95% CI of 3.5% to 22%) and was statistically significantly higher than the estimate of the general population, p < 0.001. The clinical suspicion of autism in eating disorder patients was 27.5% (95% CI of 15.6% to 42.5%) and was statistically significantly higher than the estimate of the general population, p < 0.001. The proportion of new autism diagnoses was 12.5% (95% CI of 4.9% to 25.2%) and was statistically significantly higher than the estimate of the general population, p < 0.001. Figure 1 depicts these values.

### Risk associations

Thirty of the participants had restrictive eating disorders, while 10 had non-restrictive eating disorders. No statistically significant difference was seen in the percentage of autism traits exhibited by individuals with a restrictive eating disorder (*M* = 33.750, *SD* = 30.645) and non-restrictive eating disorders (*M* = 23.750, *SD* = 22.399), *t*(38) = 0.947, *p* = 0.349).

A statistically significant association between clinical suspicion of autism and the lack of a other mood disorder was present, χ^2^(1) = 5.057, p = 0.025. None of the individuals with a clinical suspicion of autism had a diagnosed other mood disorder versus 34.5% of those with no clinical suspicion of autism.

A statistically significant association between family history of autism and developmental delays was present, χ^2^(1) = 11.317, p < 0.001. Approximately 55% of those with a family history of autism reported developmental delays versus 7% of those without a family history of autism.

### Treatment outcomes

**Fig. 2:**
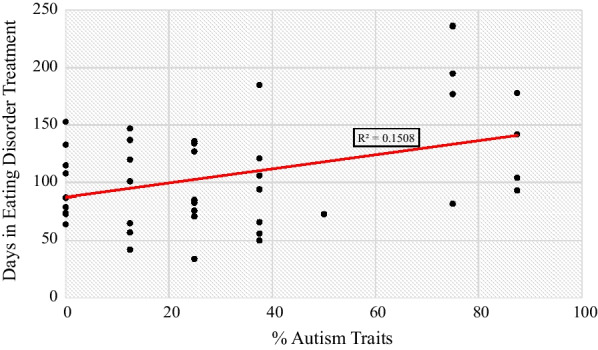
Scatterplot relationship between the percentage of autism traits and days in eating disorder treatment

A statistically significant, moderate, positive correlation between the percentage of autism traits exhibited and the number of calendar days of treatment was present, r = 0.388, p = 0.013. Approximately 15.08% of the variation in calendar days of treatment can be attributed to the percentage of autism traits exhibited by the patients. Figure 2 displays a scatterplot of the percent of autism traits and calendar days of treatment that visually depicts the relationship.

**Fig. 3:**
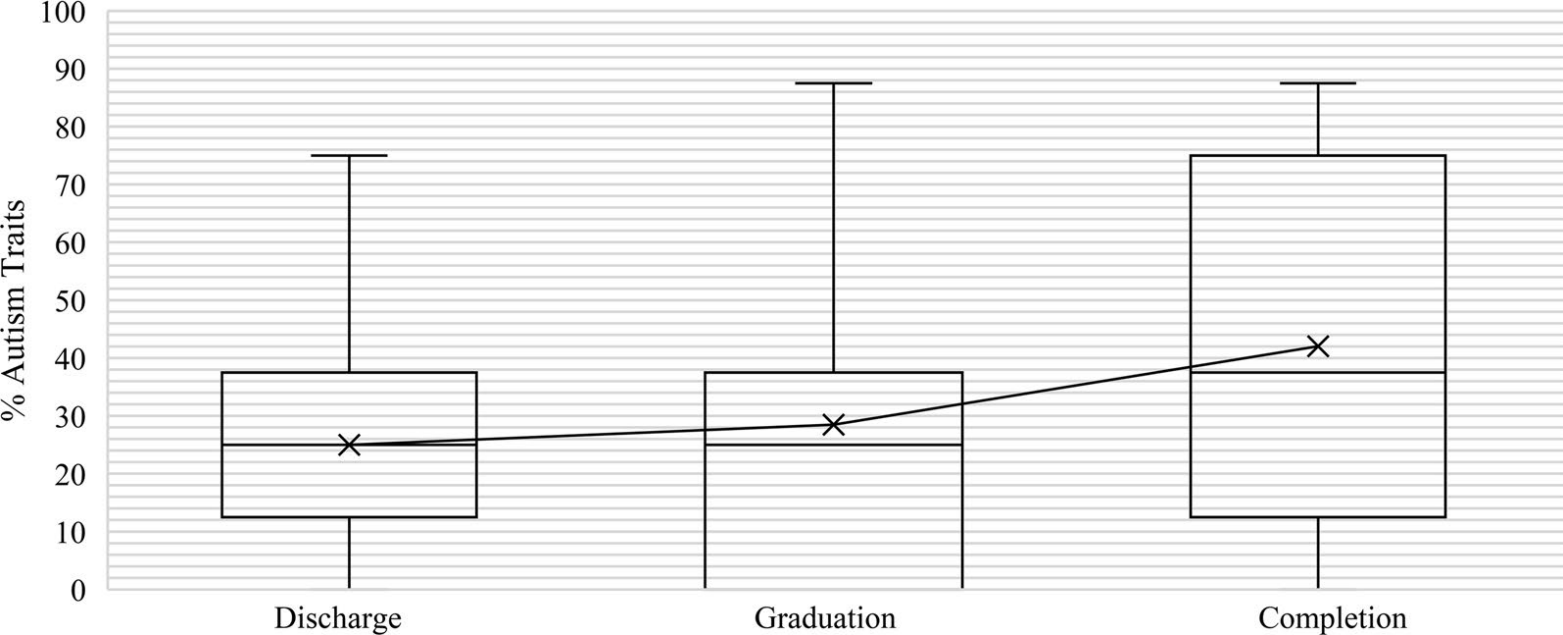
Distribution of the percentage of autism traits and treatment outcomes

Participants were classified into three groups: discharge (*n* = 11), graduation (*n* = 18) and completion (*n* = 11). The differences in the percentage of autism traits exhibited were not statistically significantly different between the groups, *p* = 0.338, and is pictured in the boxplot in Fig. 3.

## Discussion

The findings of this study support existing literature that autism is overrepresented in the eating disorder population. The CDC reports that autism is present in 1 in 116 girls (0.88%) in the United States [3]. However, upon entering the PHP level of care for an eating disorder, 10% of the study participants had a pre-existing diagnosis of autism. An additional 12.5% of study participants were given a new autism diagnosis. A total of 27.5% of participants had clinical suspicion of autism. This aligns with the previously reported autism and eating disorder co-occurrence rates of 23–32% [4, 5].

Autism traits in this study were characterized by the components of the autism diagnostic criterion outlined in the (*DSM-5-TR*) [20]. The number of autism traits that an individual possessed directly correlated with the number of calendar days in treatment, with no autism traits being associated with a mean of 98 (*SD* = 31) days in treatment and 6 autism traits associated with 173 (*SD* = 65) days in treatment. This supports the previous findings that individuals with autism have a poorer prognosis in terms of their eating disorder [5]. However, the presence of autism traits in this study did not impact whether a participant met the requirements to graduate from the eating disorder program.

The presence of autism traits was not limited to those with a restrictive eating disorder. Despite a preponderance of literature on the presence of autism in AN and avoidant-restrictive food intake disorder, autism traits have been seen in non-restrictive eating disorders [22, 23]. Additionally, none of the individuals in this study identified to have autism were diagnosed with bipolar I disorder, bipolar II disorder, disruptive mood dysregulation disorder, or cyclothymic disorder. A 7.5% cooccurrence rate of autism and bipolar disorder has been seen in adults [24]. The predominance of adolescents in this study may contribute to lack of other mood disorder diagnoses.

### Recommendations and practical applications

Further research is needed on adaptions to care for those with autism undergoing treatment for an eating disorder. However, the literature does suggest that individuals with autism are less likely to respond to cognitive behavioral therapy [14]. Neurodiversity-affirming care can also be applied to this patient population, such as avoiding sarcasm and euphemisms, providing concise verbal and written instructions, allowing the use of sensory fidgets and safe stimming, and breaking tasks down into small steps. Treatment goals should be tailored to the individual in a way that meets the individual’s needs, rather than normalizing behaviors to neurotypical social standards [18]. Returning the individual’s relationship to food or exercise to what it was before the onset of the eating disorder is a suggested measure to apply.

### Limitations

Limitations of this study are the small sample size and that the participants are from a single ED treatment center. Evaluating data from a single treatment center allows for diagnostic consistency across the sample population but is open to an internal bias of the diagnosing clinicians. However, since the sample size yielded statistically significant findings, it is worth evaluating in a larger sample across multiple treatment locations.

While this particular treatment center serves participants from the surrounding areas of eastern Washington and Oregon, it is worth noting that the economy in the region where the Recovery and Wellness Center is located is anchored in research, development, and technology [25]. More scientists and engineers per capita are located in the region than anywhere else in the nation [25]. Known as the "Silicone Valley Phenomenon," an area rich in technical professionals is linked to higher autism rates in those professionals and their children [26], however no studies have evaluated this phenomenon in this area.

## Conclusions

Eating disorder professionals in this study identified autism in 17.5% of adolescent and young adult females who entered treatment at the PHP level of care for an eating disorder who had not previously been diagnosed with autism. Recognizing undiagnosed autism may benefit the patient, their family, and the clinical team to have a greater understanding of the individual and their treatment needs. Eating disorder professionals can anticipate that individuals with autism may be in treatment for a longer duration, as supported by the increasing number of days of treatment associated with the number of autism traits.

## Data Availability

The data that support the findings of this study are available on reasonable written request to the author.

## Abbreviations

AN: Anorexia Nervosa
CDC: Centers for Disease Control and Prevention
DSM-5-TR: *Diagnostic and Statistical Manual, 5th edition, text revision*
IOP: Intensive outpatient program
PHP: Partial hospitalization program
